# Method for organotypic tissue culture in the aged animal

**DOI:** 10.1016/j.mex.2017.03.003

**Published:** 2017-04-07

**Authors:** Jared Schommer, Matthew Schrag, Alexander Nackenoff, Gurdeep Marwarha, Othman Ghribi

**Affiliations:** a Biomedical Sciences, University of North Dakota School of Medicine and Health Sciences, Grand Forks, ND, United States; b Department of Neurology, Vanderbilt University School of Medicine, Nashville, TN, United States

**Keywords:** We are describing a method for organotypic tissue culture in the aged animal in this manuscript, Organotypic slices, Aged brain, Hippocampus

## Abstract

Organotypic slicing of brain tissue from young rodents has been used as a powerful model system for biomedical research [1], [2], [3]. Organotypic slicing complements cell culture and *in vivo* studies in multiple facets. This system can be useful for investigating manipulation of cellular signaling pathways without the hindrance of the blood-brain barrier while sacrificing fewer animals in the process. It also allows for preserved cellular connectivity and local intact circuitry which is a drawback of isolated cell cultures. Studies on age-related diseases have mainly used embryonic or early postnatal organotypic slice tissue. Excluding synaptic plasticity studies that are usually carried-out over a few hours and use adult mice or rats, a handful of studies performed on adult animals have had success for survival of slices [4], [5]. Here we describe a method for culturing organotypic slices with high viability from hippocampus of aged mice and rabbits.

• Our method permits slices from mice as old as 16 months and rabbits as old as years of age to survive *ex vivo* up to 8 weeks [6], [7], [8], [9]. Such a slice system may be relevant to investigating age-related brain diseases.

## Graphical abstract

**Figure:**
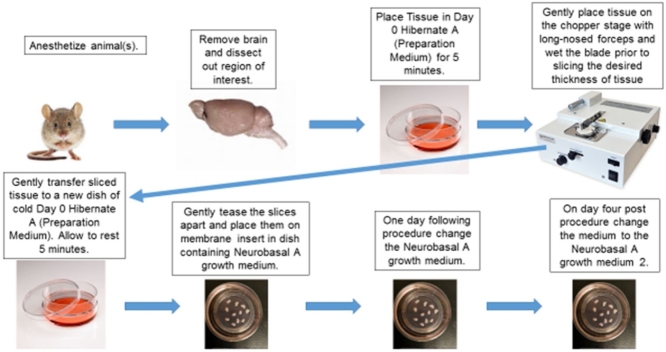

## Method details

### Materials

**Table:** 

Material	Company	Catalog Number
McIlwain Tissue Chopper	The Mickle Laboratory Engineering Co. LTD.	Model MTC/2
Teflon insert	The Mickle Laboratory Engineering Co. LTD.	
Grade 50 hardened filter paper	Whatman	1450-055
35 × 15 mm tissue culture treated dishes	Santa Cruz	Sc-200284
100 × 20 mm cell culture dishes	Greiner Bio-One	664-160
Size 2 oil paint brushes	Silver Fox	
Long-nosed forceps		
Premium Sterile Stainless Steel Scalpel Blades – #22	Havel’s	FHS22
0.4 μm, 30 mm cell culture inserts	Millipore	PICMORG50
Hibernate A	Brain Bits	Hibernate A
l-Glutamine 200 mM (100×)	Gibco	25030-081
Horse Serum	Gibco	16050-122
Antibiotic/Antimycotic (100×)	Gibco	15240-062
Neurobasal-A Medium	Gibco	10888-022
2% B27 Supplement (50×)	Gibco	17504-044

### Method

Preparation—Prior to animal sacrifice

Day 0 Medium Preparation

Hibernate A (preparation medium):

**Table:** 

To a sterile 50 mL centrifuge tube add:
0.5 mM Glutamine (250 μL of stock solution)
10 mL Horse Serum
40 mL standard Hibernate A Medium

**Fig. 1:**
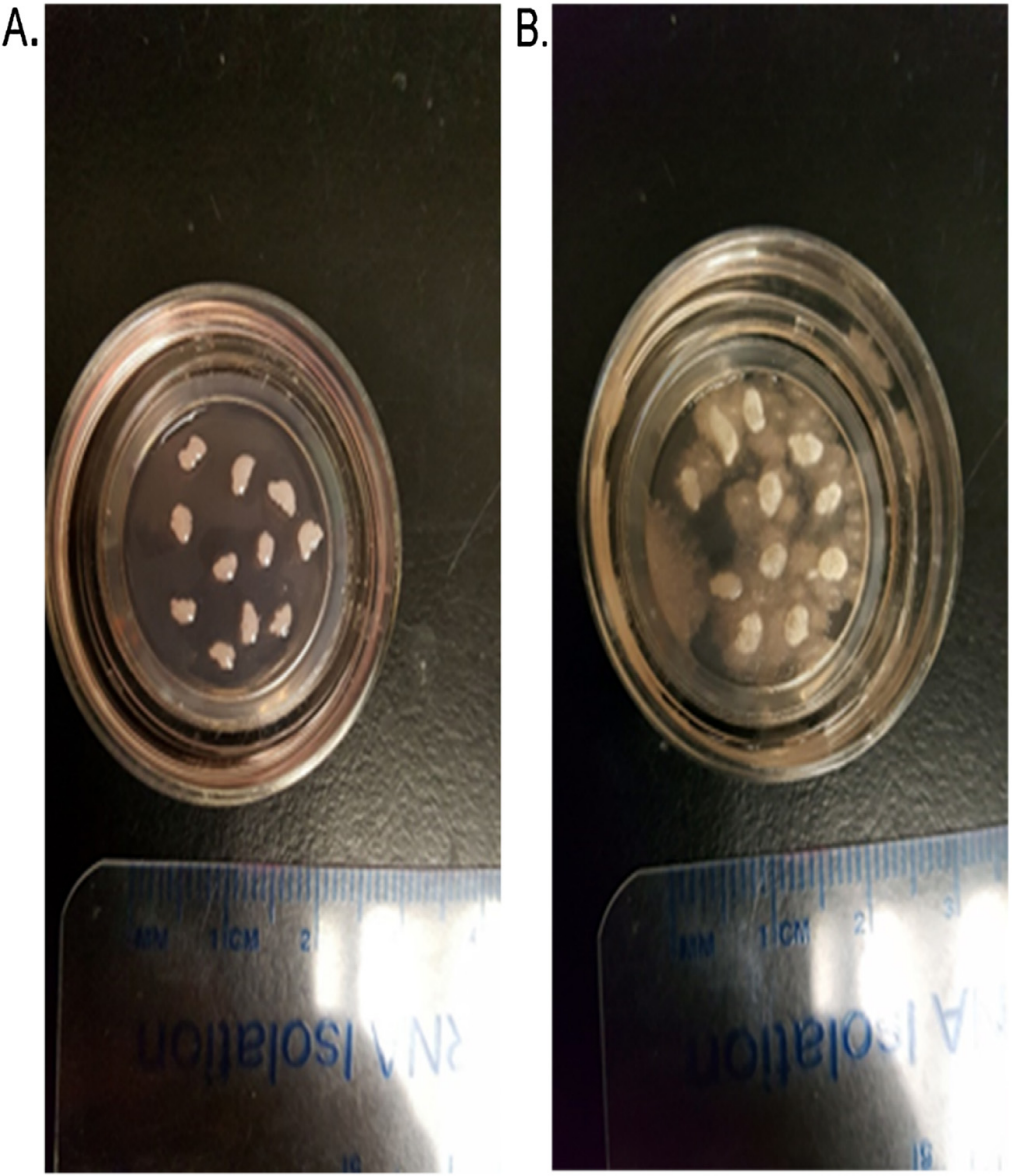
Hippocampal slices from 1 year old C57BL6 mice. A. Healthy dish of mouse hippocampal slices 10 days post tissue sectioning. B. Dead/Dying infected mouse hippocampal slices 10 days post tissue sectioning.

**Fig. 2:**
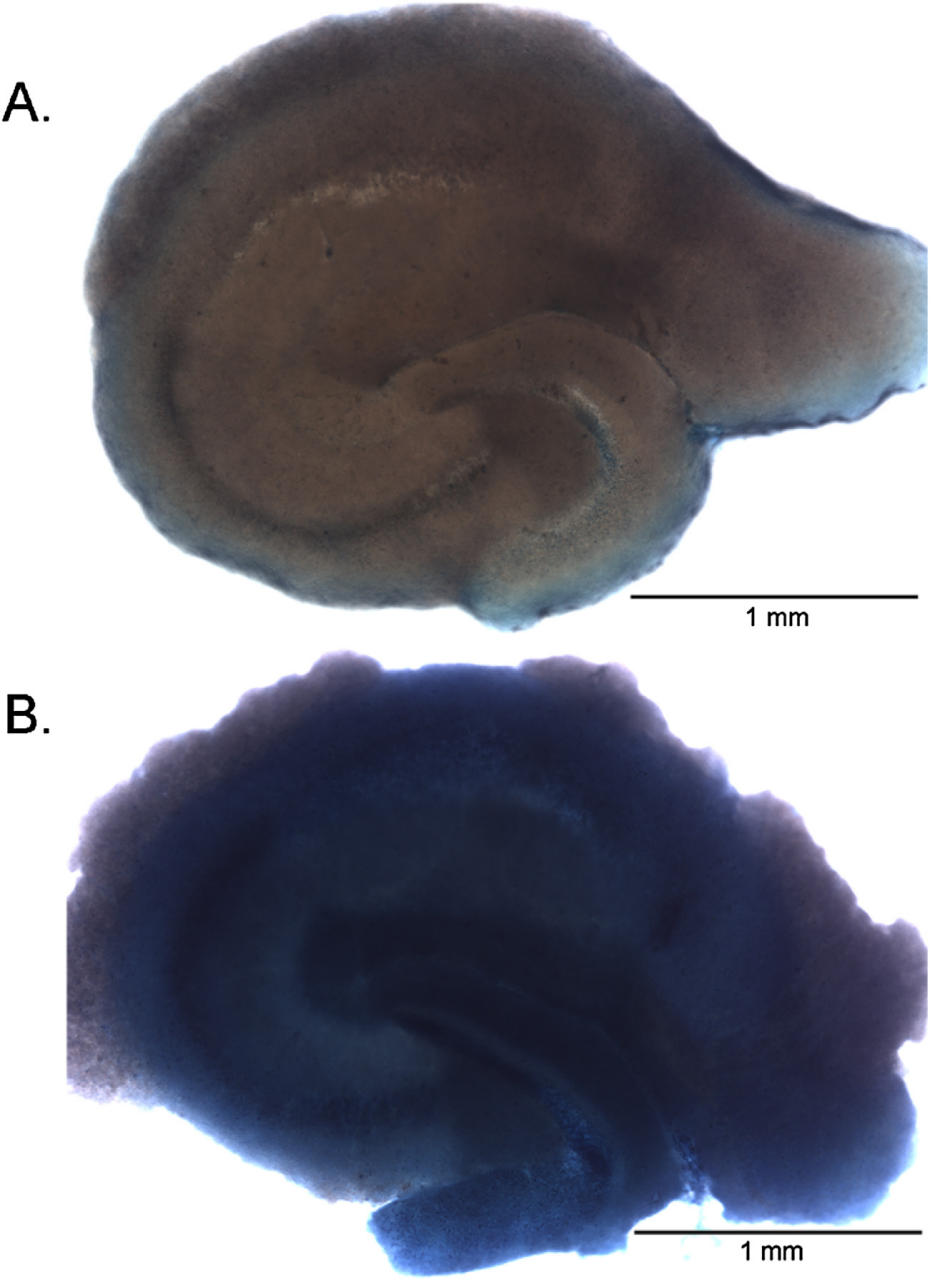
Hippocampal slices from 10.5 month old B6129SF2/J mice. A. Healthy hippocampal slice 7 days post tissue sectioning exposed to Trypan Blue staining. B. Medium deprived dead hippocampal slice 7 days post tissue sectioning exposed to Trypan Blue staining.

**Fig. 3:**
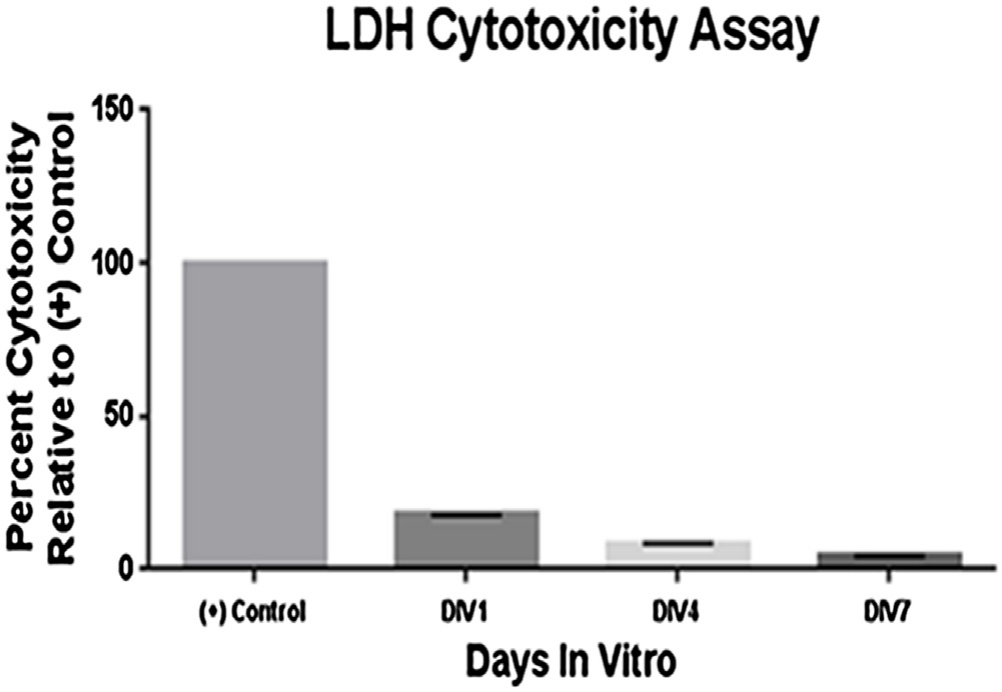
LDH Assay on the medium of culture dishes containing 4 hippocampal slices of 1 year old C57BL6 mice at sequential days *In vitro.*

Prepare 2–3 batches if you desire extra medium and/or to change out when medium containing the slices starts to discolor (Fig. 1, Fig. 2, Fig. 3 ).

Neurobasal A (growth medium):

**Table:** 

To a sterile 50 mL centrifuge tube add:
20% Horse Serum (8 mL)
400 μL standard antibiotic mixture (Antibiotic/Antimycotic)
40 mL Neurobasal A Medium

Prepare Day 1 and Day 4 – Treatment Day Medium fresh on the day of use

**Table:** 

****Day 1****	****Day 4 through Treatment Day****
Neurobasal A (growth medium 1):	Neurobasal A (growth medium 2):
To a sterile 50 mL centrifuge tube add:	To a sterile 50 mL centrifuge tube add:
20% Horse Serum (8 mL)	2% B27 supplement (800 μL)
400 μL standard antibiotic mixture (Antibiotic/Antimycotic)	400 μL standard antibiotic mixture (Antibiotic/Antimycotic)
40 mL Neurobasal A Medium	40 mL Neurobasal A Medium

*McIlwain Chopper Preparation*:

• Prepare the chopper by adjusting the dial for the desired slice thickness (we have used 250 μM and 300 μM slices).
• Install a sharp double-sided razor and loosely attach the clamp.
• Thoroughly clean the stage of the chopper and blade with 70% ethanol.
• Place a sterile Teflon insert surrounded by 2 filter paper disks on the stage.
• Turn the dial on the chopper to allow the arm to drop onto the stage containing the Teflon insert and filter papers. Once the arm has dropped make sure the blade is resting flush on top of the stage, then tighten the clamp.
• Just prior to use, wet the top filter paper with a few drops of Hibernate A preparation medium and wet the blade using the paintbrush to ensure that the tissue will stick to the filter paper but not the blade.

*Insert Preparation*:

• Place 1.1 mL of growth medium 1 into the desired number of 35 mm tissue culture dishes. For hippocampal slices from mice, you can expect to use 3 dishes per mouse (8–10 slices per dish). For hippocampal slices from rabbit you can expect to use 12–15 dishes per rabbit (4–6 slices per dish).
• Place one Millicell insert in each dish trying to avoid trapping air bubbles underneath the membrane to allow the tissue to contact the medium.
• Store the prepared dishes in the incubator (35 °C, 5% CO_2_) for at least 1 h prior to use.

*Procedure*:

• Anesthetize animal with Euthasol diluted 1:1 with dH_2_O and rapidly decapitate. Other forms of anesthesia also work including CO_2_ and Ketamine/Xylazine.
• Dissect area of interest and place in chilled preparation medium in a 100 mm tissue culture dish. Store on ice for 5 min or less.
• Transfer the tissue to the stage of the McIlwain chopper and proceed to chop the tissue.
• Gently move the sliced tissue from the stage into a new 100 mm tissue culture dish containing chilled preparation medium and allow the slices to sit in the solution for 5 min.
• Transfer to a new 100 mm tissue dish containing 4 mL of chilled preparation medium. Less medium in the dish allows for easier handling and separation of slices.
• Gently tease the slices apart using a small size 2 oil paint brush and scalpel. Once separated, pull the slices from their outer extremity onto the scalpel blade using the paint brush while being careful not to damage the slices integrity. Transfer the slices from the scalpel blade to the membrane of the dishes that were prior placed in the incubator using the paint brush again on the outer extremity of the slice to minimize damage to the slice. Each membrane can hold 8–10 mouse hippocampal slices or 4–6 rabbit hippocampal slices.
• Change the medium on Day 1 and on every third day. Do this as quick as possible, if necessary only change media on 2–4 dishes at a time.

The sections attach to the culture membranes in a few days and become fully attached to the membrane after ten days. One half of the growth medium should be replaced every 3–4 days. Sections plated at lower density (*i.e*. 3–5 sections of mouse hippocampus per membrane) will require media exchange every 7–10 days.

Though infection is rare (roughly 1 in 50 dishes) and user dependent, standard antibiotic mixture is used to minimize infection throughout the duration of culture. If desired, user may exclude standard antibiotic mixture following day 4 with similar infection rate.

## Funding

This article was funded by NIH (Grant number: NIH-RO1AG0145264) to Dr. Othman Ghribi.
